# Clinical perspectives on the menstrual pictogram for the assessment of heavy menstrual bleeding

**DOI:** 10.1093/hropen/hoac048

**Published:** 2022-10-29

**Authors:** Sukhbir S Singh, Joaquin Calaf Alsina, Silvia Vannuccini, Kaori Koga, Agnaldo Lopes Silva-Filho, Xin Yang, Jean-Philippe Estrade, William Catherino

**Affiliations:** University of Ottawa & Ottawa Hospital Research Institute, Ottawa, Ontario, Canada; Hospital de la Santa Creu i Sant Pau, Universitat Autónoma, Barcelona, Spain; Careggi University Hospital, Florence, Italy; University of Tokyo, Tokyo, Japan; Universidade Federal de Minas Gerais, Belo Horizonte, Brazil; Peking University People’s Hospital, Beijing, China; Clinique Bouchard-Elsan, Marseille, France; Uniformed Services University of the Health Sciences, Bethesda, MD, USA

**Keywords:** heavy menstrual bleeding, menstrual blood loss, uterine fibroids, menstrual pictogram, alkaline hematin method, abnormal uterine bleeding

## Abstract

Heavy menstrual bleeding (HMB) has an estimated prevalence of 18–32% but is known to be under-reported due to poor recognition and estimation of menstrual blood loss (MBL). HMB can negatively impact quality of life, affecting social interactions, work productivity and sexual life. Abnormal menstrual bleeding may have an underlying structural or systemic cause, such as endometrial and myometrial disorders; however, for some, there is no identified pathological cause. Several methods are available for assessing MBL, including the alkaline hematin (AH) method and the menstrual pictogram (MP). The AH method is considered to be the most accurate way to monitor MBL; however, it is associated with inconvenience and expense, therefore limiting its value outside of research. The MP requires the user to select an icon from a chart that reflects the appearance of a used sanitary product; the icon is associated with a blood volume that can be used to determine MBL. Validation studies have demonstrated that the results of the MP and AH method are well correlated, showing that the MP can measure MBL with sufficient accuracy. Additionally, the MP is more convenient for users, less expensive than the AH method, may be used in regions where the AH method is unavailable and may also be used as part of a digital application. Overall, the MP offers a convenient approach to monitor MBL both in research and clinical practice settings.

## Introduction

Heavy menstrual bleeding (HMB) is defined as blood loss of >80 ml per menstrual cycle (Hallberg *et al.*, 1966), or excessive menstrual blood loss (MBL) that interferes with a woman’s physical, emotional, social and material quality of life (QoL). HMB can occur alone or with other symptoms (National Institute for Health and Care Excellence, 2018).

### HMB is under-recognized and under-reported

There are sparse data on HMB global prevalence in the general population, although a prevalence of 18–32% at any time during reproductive life has been suggested. This can vary across regions according to sample population characteristics (e.g. parity, age) and is dependent on HMB definition (Karlsson *et al.*, 2014; Fraser *et al.*, 2015, Hapangama and Bulmer, 2016; Ding *et al.*, 2019).

HMB is known to be extensively under-reported, and potentially only 6% of women with HMB seek medical help (Fraser *et al.*, 2015). This is largely due to inaccurate individual self-perception of HMB (Magnay *et al.*, 2018) and normalization of symptoms (Fraser *et al.*, 2015). For example, one population study demonstrated poor correlation between women’s perception of and actual MBL: 37% and 4% of women with blood loss >80 ml considered their MBL to be moderate or scanty, respectively; 14% of women with blood loss <20 ml considered their MBL to be heavy (Hallberg *et al.*, 1966). This is supported by studies including self-reported data, which report a lower HMB prevalence than primary clinical studies (Fraser *et al.*, 2015). HMB recognition is also influenced by cultural factors (Edelman *et al.*, 2007; Bitzer *et al.*, 2013): menstrual taboos can promote a culture of silence (Harlow and Campbell, 2004) and some cultures consider menstrual blood to be ‘medically cleansing’ or a sign of health (Bitzer *et al.*, 2013).

The International Federation of Gynecology and Obstetrics Working Group on Menstrual Disorders developed the PALM-COEIN (polyp; adenomyosis; leiomyoma; malignancy and hyperplasia; coagulopathy; ovulatory dysfunction; endometrial; iatrogenic; and not yet classified) classification system for causes of abnormal uterine bleeding (AUB), defined as menstrual bleeding that is abnormal in duration, volume and/or frequency for ≥3 months. The term AUB also encompasses HMB (Munro *et al.*, 2011). Although, for some women, there is no obvious pathological cause of their AUB, others may experience one or more entities that can cause or contribute to AUB, including structural causes (polyps, adenomyosis, leiomyoma [fibroids], malignancies) or non-structural causes (coagulation, ovulatory, endometrial iatrogenic disorders) (Munro *et al.*, 2011; Hapangama and Bulmer, 2016; Cheong *et al.*, 2017). Depending on severity, HMB may also lead to anemia (Borah *et al.*, 2013; Stewart *et al.*, 2013; Salehi *et al.*, 2015; David *et al.*, 2016; Soliman *et al.*, 2017). It is therefore clear that under-reporting of HMB is of concern and improving identification methods may lead to timely diagnosis and treatment options.

### HMB negatively impacts QoL

HMB has been shown to adversely affect QoL, including impacting physical activities, social interactions, work productivity, well-being and sexual life (Lukes *et al.*, 2012; Gokyildiz *et al.*, 2013; Su *et al.*, 2020). It is the impact on daily life that will often lead individuals to seek care from their healthcare providers (Lukes *et al.*, 2012).

*Post-hoc* analysis of data from two randomized, placebo-controlled studies of an oral tranexamic acid formulation in women with HMB revealed that higher daily MBL was associated with worse ratings of health-related QoL (Menorrhagia Impact Questionnaire) (Lukes *et al.*, 2012). Data from both a case–control study and a Swedish cross-sectional study found that scores on all eight domains of the Short Form-36 QoL scale (physical functioning, physical role, pain, general health, vitality, social role functioning, emotional role functioning and mental health) were significantly lower in women who reported HMB than in women in the control group (either relatives of the participants without any specific health problems or women with normal MBL) (Gokyildiz *et al.*, 2013; Karlsson *et al.*, 2014). To improve QoL in women with HMB, efforts to assess and reduce MBL should be a priority for healthcare providers (Lukes *et al.*, 2012).

This report presents the advantages and limitations of the most commonly used methods for MBL assessment, with a focus on the potential use of the menstrual pictogram (MP), a tool with relevance for both research and routine clinical practice, compared with the alkaline hematin (AH) method.

## Quantitative assessment of MBL

### AH method

The AH method was established for the quantitative assessment of MBL and is considered to be the ‘gold standard’ in terms of accuracy (Wyatt *et al.*, 2001; Magnay *et al.*, 2018). Based on current United States Food and Drug Administration guidance, the AH method is typically used for the diagnosis and assessment of HMB in research settings (Magnay *et al.*, 2018). The AH method was developed >50 years ago and involves chemical extraction of hemoglobin from used sanitary products. It was initially validated for use with cotton-based sanitary products and blood recovery was 96% after a 20 h incubation. Following protocol modifications to simplify and improve the speed (Wyatt *et al.*, 2001), the efficiency of blood extraction from a selection of sanitary products ranged from 75 to 107% (Magnay *et al.*, 2018).

More than a decade ago, most sanitary towels (also referred to as sanitary pads) contained cotton as the main component of the absorbent core, whereas today, the majority of products contain superabsorbent polymer (SAP) granules (Magnay *et al.*, 2014; Woeller and Hochwalt, 2015; P&G (Proctor and Gamble), 2019). The AH method was subsequently adapted and revalidated for use with a selected brand of SAP-containing towels (Magnay *et al.*, 2011); recovery of at least 90% (≥85% with automation) of simulated menstrual fluid volumes was observed (Magnay *et al.*, 2018).

However, the AH method requires women to collect and send used sanitary products for laboratory analysis, which presents some challenges: it can be impractical and inconvenient, and it requires laboratory expertise and costs to interpret and report results (The Menorrhagia Research Group *et al.*, 2004; Schumacher *et al.*, 2012; Magnay *et al.*, 2014, 2018). The AH method is subject to incomplete patient compliance and collection variability, including variation in sanitary products, with associated variability in recovered amount of AH and requirement for calibration curves for each product (El-Nashar *et al.*, 2015). Underestimation of blood loss due to overflow from the sanitary product is exacerbated by non-blood components not being detected by the AH method (Fraser *et al.*, 1985; Fraser *et al.*, 2001; Wyatt *et al.*, 2001; The Menorrhagia Research Group *et al.*, 2004; Magnay *et al.*, 2014, 2018).

These practical limitations prevent the AH method from being used beyond research settings. Furthermore, women may be deterred from participating in clinical trials and complying with the study requirements, due to the inconvenience of having to collect, store and send used sanitary wear (Magnay *et al.*, 2018).

### Pictorial methods

As the focus of treatment must be the improvement of women’s symptoms and QoL (Mohan *et al.*, 2007; Cheong *et al.*, 2017), it follows that the AH method of quantifying MBL has less relevance in clinical practice. Pictorial methods of measuring MBL, such as the original pictorial blood loss assessment chart (PBAC) and MP, are simple, quick to use, semi-quantitative, patient-reported outcome tools used to determine MBL volume utilizing icon-based visual scoring systems for commonly used sanitary products (Higham *et al.*, 1990; Janssen *et al.*, 1995; Wyatt *et al.*, 2001; Magnay *et al.*, 2013, 2014). These tools may be beneficial in both adult and adolescent patients and are particularly useful for monitoring treatment response (Mohan *et al.*, 2007; Magnay *et al.*, 2018).

Unfortunately, there are important drawbacks to the currently used pictorial methods, including variable sensitivity and specificity versus the AH method, due to only three icons being used, contributing to reduced accuracy. The MP also requires a paper diary record for every used sanitary towel or tampon, a limitation associated with decreased accuracy (Magnay *et al.*, 2013, 2018; El-Nashar *et al.*, 2015). There is, therefore, an unmet need for an accurate, semi-quantitative method of MBL assessment that is acceptable for use in clinical trials and clinical practice.

The original PBAC and MP were validated for use with the cotton-containing sanitary products that were available more than a decade ago (Janssen *et al.*, 1995; Wyatt *et al.*, 2001; Magnay *et al.*, 2018), and have subsequently been revalidated with SAP-containing products that are now commonly used (although validation has only been conducted for a limited number of current products) (Magnay *et al.*, 2014, 2018). The revalidated PBAC and MP still both have the disadvantage of women having to recall/record results (Magnay *et al.*, 2018); however, although the MP (which has the advantage of estimating MBL in milliliters and being directly comparable with the AH method) (Magnay *et al.*, 2018) can differentiate between sanitary product absorbency ratings, the PBAC (which uses a scoring system that is proportional, but not equivalent to MBL) (Magnay *et al.*, 2018) cannot. Furthermore, the PBAC has been shown to overestimate MBL in some women, thereby limiting its value in clinical practice (Magnay *et al.*, 2014., 2018). An overview of some of the recent original and revalidated PBAC and MP data, highlighting sensitivity and specificity, and correlation with the AH method is provided in Table I. A full review of the currently available data for these, and other methods used to measure MBL, has been published (Magnay *et al.*, 2018).

**Table I:** Overview of pictorial blood-loss assessment chart and menstrual pictogram data for assessing menstrual blood loss in clinical trials.^a^

	PBAC	MP
Sensitivity and specificity	Diagnosis of MBL >80 ml (n = 950) (Higham *et al.*, 1990; Deeny and Davis, 1994; Janssen *et al.*, 1995; Barr *et al.*, 1999; Reid *et al.*, 2000; Zakherah *et al.*, 2011; Hald and Lieng, 2014): Sensitivity: 58–99% Specificity: 7.5–89% Diagnosis of self-perceived HMB Sensitivity: 78.5% Specificity: 75.8% (n = 429) (Hald and Lieng, 2014)	Diagnosis of MBL >80 ml (Wyatt *et al.*, 2001; Magnay *et al.*, 2014) or identifying ≥50% decrease in MBL (n = 314) (Larsen *et al.*, 2013): Sensitivity: 82–96% Specificity: 88–94%
Predictive value	Predictive value of diagnosing HMB with a PBAC score cut-off of 100 (n = 103) (Reid *et al.*, 2000): PPV: 62% NPV: 60% Predictive value of diagnosing HMB with a modified PBAC score cut-off of 185 (n = 288) (Janssen *et al.*, 1995): PPV: 85.9% NPV: 84.8% Predictive value of diagnosing HMB with a complaint of heavy MBL (n = 288) (Janssen *et al.*, 1995): PPV: 55.9%	Predictive value of diagnosing HMB (n = 170) (Larsen *et al.*, 2013): PPV: 91% NPV: 83%
Correlation with MBL assessed by AH method	Moderate-to-high (*r *=* *0.466–0.847) correlation with MBL or change in MBL from baseline assessed by the AH method (n = 328) (Higham *et al.*, 1990; Reid *et al.*, 2000; Zakherah *et al.*, 2011)	High (*r *=* *0.81–0.86) correlation with MBL or change in MBL from baseline (n = 206) (Larsen *et al.*, 2013; Magnay *et al.*, 2014)

a Data shown are for different versions of the MP and PBAC, which may have been adapted for use for study purposes. A full review of these data has been published (Magnay *et al.*, 2018).

PBAC, pictorial blood-loss assessment chart; MP, menstrual pictogram; MBL, menstrual blood loss; HMB, heavy menstrual bleeding; AH, alkaline hematin; PPV, positive predictive value; NPV, negative predictive value.

## Development and validation of the MP

The revalidated MP (for use with SAP-containing sanitary products; hereafter referred to as the MP) is a later modification of the PBAC developed to assess MBL in clinical trials (Magnay *et al.*, 2014). The MP allows women to assess the visual appearance of used sanitary products (Magnay *et al.*, 2013, 2014), and the pictograms are used to provide an estimation of MBL (Magnay *et al.*, 2013).

**Figure 1.**
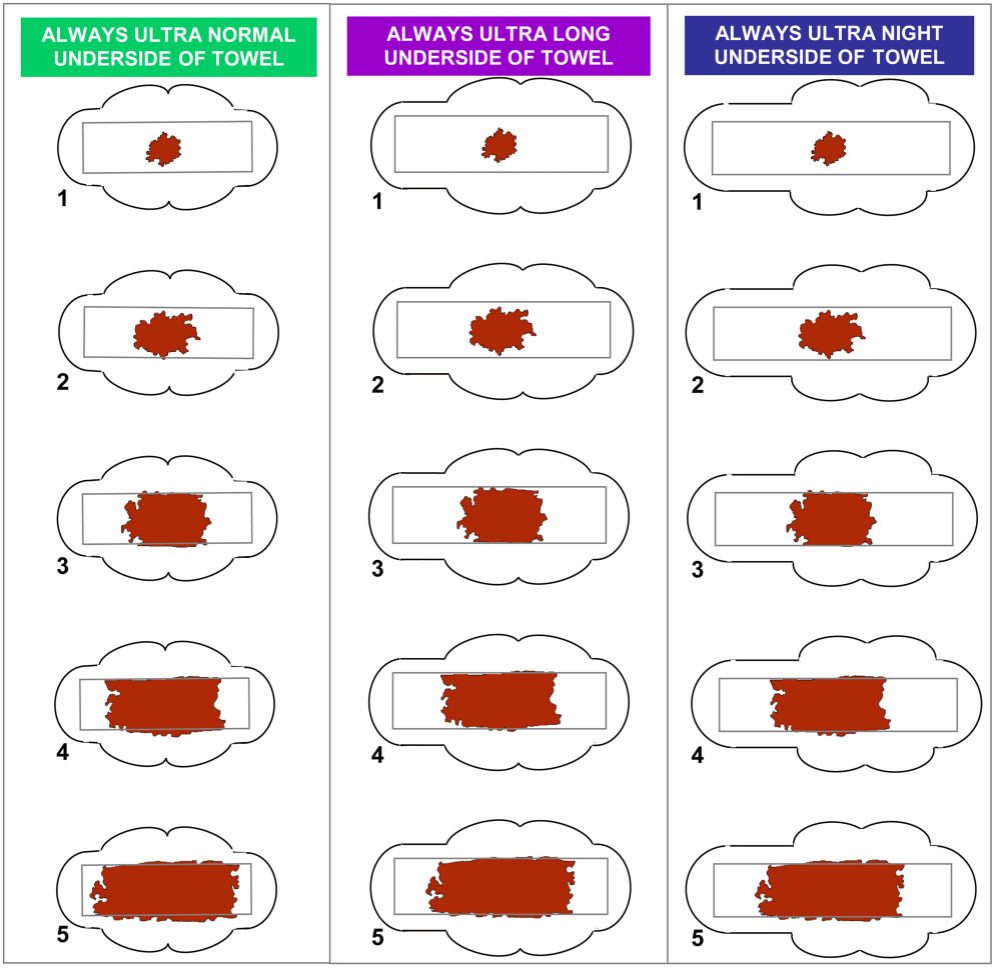
**The revalidated menstrual pictogram.** The menstrual pictogram requires women to assess their sanitary product upon changing, by selecting the image that looks the most like the underside of their sanitary product. In Magnay *et al.* (2014), blood loss (ml) was assigned to each pictogram: 0.5, 1.5, 4, 6.5 and 12.5 for icons 1–5 of the ‘normal’ sanitary products, 0.5, 1.5, 3.5, 6.5 and 12.5 for icons 1–5 for the ‘long’ sanitary products, and 0.5, 2, 4.5, 8 and 14 for icons 1–5 of the ‘night’ sanitary products. Reprinted with permissions from Magnay *et al.* (2014).

The MP comprises diagrams, with five icons that depict a graded series of stained towels or tampons (Fig. 1); each icon is associated with a blood volume derived from measurements taken by the AH method (Magnay *et al.*, 2013, 2014). Women are asked to complete the MP whenever a sanitary product is changed, by choosing a pictogram icon that corresponds with the degree of staining on the underside of the sanitary product (Magnay *et al.*, 2014).

**Figure 2.**
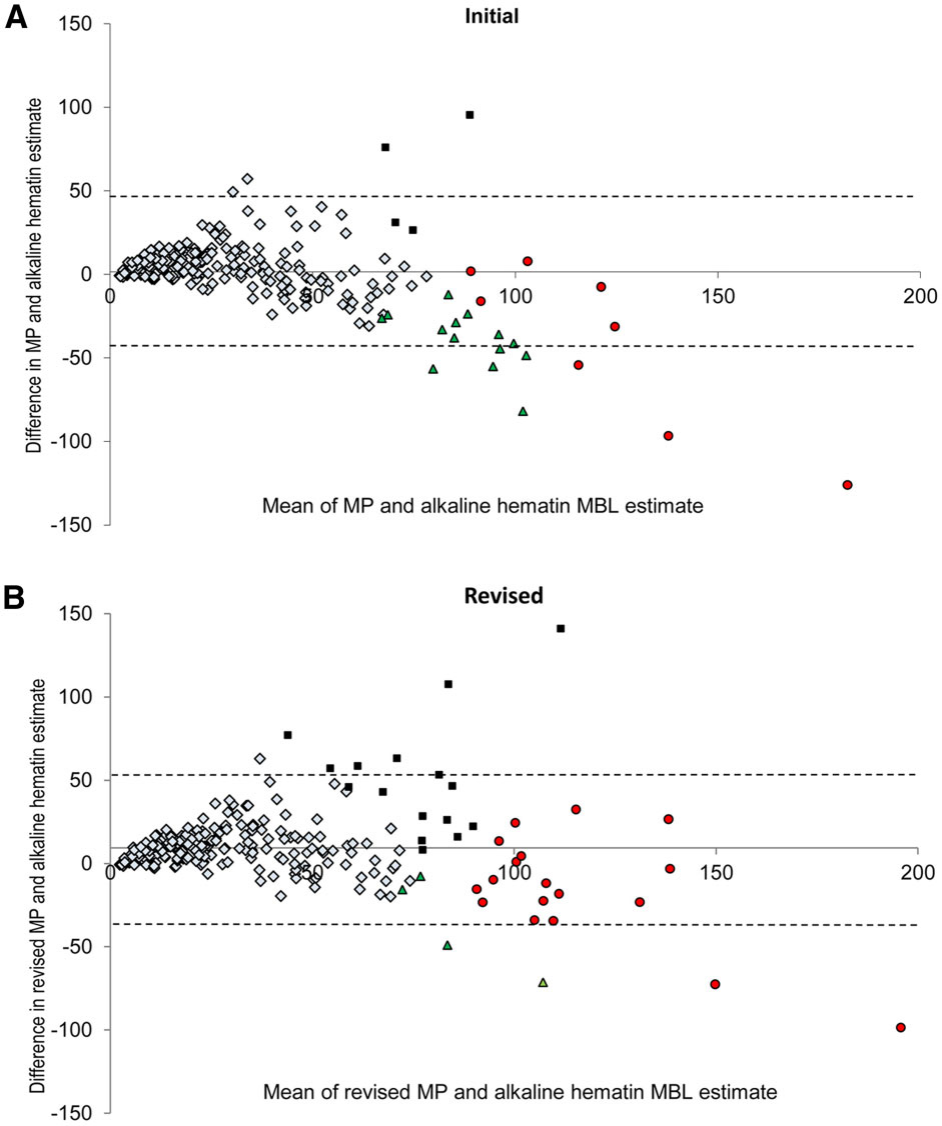
**Bland–Altman analysis of menstrual blood loss based on participant estimates with the menstrual pictogram versus alkaline hematin method.** Bland–Altman analysis of participant revalidated menstrual pictogram (MP) estimate of menstrual blood loss (MBL) versus alkaline hematin estimate of menstrual blood loss (**A**) before and (**B**) after revision of icon blood volume, in Magnay *et al.* (2014). Symbol interpretation: light blue diamond = true negative; red circle = true positive; green triangle = false negative; black square = false positive. Dotted lines indicate 95% limits of agreement. Reprinted with permissions from Magnay *et al.* (2014).

In the validation study of the MP for measuring MBL (with the AH method as the reference standard) (Magnay *et al.*, 2014), the median blood loss for the 22 HMB cycles was 111 ml (range, 80.1–245 ml). In contrast, the median blood loss for the 213 normal cycles was 17 ml (range: 1–80 ml). Information on patient compliance/adherence was not reported. Overall, of 3325 sanitary towels collected, only 10 were excluded from analysis due to missing participant icon data (Magnay *et al.*, 2014). Following correction for the incremental rise in blood fraction with volume, the MP demonstrated high sensitivity (82% [participant assessments identified 18/22 HMB cycles as >80 ml]) and specificity (92% [197/213 normal cycles were identified as ≤80 ml]) in diagnosing HMB. Figure 2 shows the Bland–Altman analysis of participant MP estimate of MBL versus AH estimate of MBL, after revision of icon blood volume. The expert ratings revealed a sensitivity of 95% (21/22 HMB cycles) and a specificity of 89% (190/213 normal cycles). Furthermore, AH and MP scores were significantly correlated (*r *=* *0.81, *P* < 0.0001) (Magnay *et al.*, 2014).

A second validation study for a further adapted version of the MP (MPv3) has been published (Haberland *et al.*, 2020). The MPv3 (on an electronic device given to patients) was included alongside the AH method in a Phase 2 study of a novel medical treatment for uterine fibroids. Comparison and quantitative assessment of the MPv3 was performed based on participant use of sanitary pads or tampons (Haberland *et al.*, 2020). Full details of comparisons and statistical analyses have been described (Haberland *et al.,* 2020). The results demonstrated that bleeding outcomes measured by the MPv3 strongly correlated with those from the AH method. Outcomes were determined by intraclass correlation coefficients (ICCs) for reliability of the MPv3 to provide reproducible scores over time (test–retest), correlation coefficients for the extent to which MBL measured by MPv3 is related to observed MBL (criterion validity) and responsiveness (Haberland *et al.*, 2020). Correlation coefficients showed a strong association between the MPv3 and the AH method with regard to test–retest reliability (ICC estimate [95% CI] of 0.93 [0.88–0.96] during screening and randomization periods, and 0.96 [0.94–0.97] during treatment in AH-defined stable women); criterion validity (*r_s_* = 0.72 at randomization and *r_s_* = 0.97 at end of treatment); and responsiveness (*r_s_* = 0.86 for change in monthly sum scores) (Haberland *et al.*, 2020). There was also a lower frequency of missing data for the MPv3, versus the AH method, indicating improved compliance with the MPv3—a key benefit (Haberland *et al.*, 2020).

Overall, currently available evidence suggests that the MP and MPv3 meet the unmet need for more accurate and patient-friendly methods for quantitative MBL evaluation, potentially supporting improved clinical care and more informed decision-making. Although all methods for the assessment of MBL have limitations, pictorial methods (especially the MP/MPv3) offer a good balance between ease of use and validated accuracy (Magnay *et al.*, 2018).

## Clinician opinion of the MPv3 and opportunities for future use

The MPv3 is simple, user-friendly and inexpensive, and has demonstrated responsiveness and reliability during validation for use in clinical trials evaluating MBL (Magnay *et al.*, 2014; Haberland *et al.*, 2020). MP data are available from a number of studies (Wyatt *et al.*, 2001; Wyatt *et al.*, 2002; Larsen *et al.*, 2013; Magnay *et al.*, 2013, 2014) and, similar to the AH method, the MPv3 can assess change in MBL over time to determine treatment response, as demonstrated in women with uterine fibroids (Haberland *et al.*, 2020). Advantages of the MPv3 over the AH method include:

- it is easy to use (Magnay *et al.*, 2014; Haberland *et al.*, 2020),
- it does not require women to mark, label, store and send used sanitary products for analysis (The Menorrhagia Research Group *et al.*, 2004; Magnay *et al.*, 2018),
- it does not require a clinic to receive, store and analyze used sanitary products (The Menorrhagia Research Group *et al.*, 2004),
- it is used as part of a digital application, making recording data straightforward, and allowing data to be easily entered alongside electronic medical records without a paper diary record for every used sanitary towel or tampon (Haberland *et al.*, 2020),
- it can be used in areas where the AH method is not available and
- it is of lower cost (Schumacher *et al.*, 2012; Magnay *et al.*, 2014).

Positive results support an opportunity for using the MPv3 to monitor treatment response for HMB-associated conditions in clinical practice. Based on the convenience and ease of use versus the AH method, the MPv3 may support patient recruitment and retention in clinical trials, and potentially improve compliance and increase the accuracy of reported results, ultimately facilitating research (Haberland *et al.*, 2020). One drawback of paper pictorial methods is that patients must record details of used sanitary products in a paper record; when used as a digital application (ideally available on any mobile device), physician and patient access to the MPv3, along with data storage and sharing, would be key advantages. Although it should be noted that not all patients may have access to a phone, be able to download the digital application or readily have access to an internet connection.

Beyond clinical trials, the MPv3 may be a valuable diagnostic tool for HMB. Reports indicate that HMB is under-reported and under-recognized, underlining the importance of de-normalizing this pathological condition (Fraser *et al.*, 2015). The MPv3 may also educate women on whether their MBL volume is abnormal and if it may indicate an underlying condition. It would allow physicians and women to gauge the severity of HMB and facilitate personalized bleeding management, as well as evaluate the efficacy of treatment of any underlying condition. Increasing awareness of HMB will, in turn, improve knowledge around its impact on reproductive health, enabling the identification and management of any adverse effects on QoL and fertility.

There is also a need to shift regional and cultural views around HMB (Harlow and Campbell, 2004; Edelman *et al.*, 2007; Bitzer *et al.*, 2013), making this a topic that women feel comfortable and confident discussing. Tools such as the MPv3 may help to empower women to openly discuss HMB with their physician. Self-reporting of symptoms and outcomes is strongly encouraged by many physicians, and helps women to play a more active role in their diagnosis and treatment.

## Conclusion

The MPv3 menstrual loss evaluation tool has been validated for the assessment of HMB and offers several opportunities for use both in research and clinical practice to evaluate treatment response and disease progression/patient follow-up. Based on these benefits, and its advantages over the AH method, the MPv3 has the potential to broaden the perception and awareness of HMB and its associated pathologies in women and clinicians, resulting in improved outcomes for these women.

## Data Availability

No new data have been generated or analyzed in support of this publication.
